# Highlights of the 1st IEEE Symposium on Biological Data Visualization

**DOI:** 10.1186/1471-2105-13-S8-S1

**Published:** 2012-05-18

**Authors:** Jessie Kennedy, Jos Roerdink

**Affiliations:** 1 Institute for Informatics & Digital Innovation, Edinburgh Napier University, UK; 2 Johann Bernoulli Institute for Mathematics and Computer Science, University of Groningen, The Netherlands

## 

This Supplement to BMC Bioinformatics contains a collection of extended articles providing highlights from the 1st IEEE Symposium on Biological Data Visualization (BioVis 2011, http://www.biovis.net) held on October 23-24, 2011, in conjunction with IEEE Visweek in Providence, RI.

The rapidly expanding field of biology creates enormous challenges for computational visualization techniques for enabling researchers to gain insight from large and highly complex data sets. The goal of the BioVis 2011 symposium was to create the premier international and interdisciplinary event for all aspects of visualization in biology. The symposium brought together researchers from the visualization, bioinformatics, and biology communities with the purpose of educating, inspiring, and engaging visualization researchers in problems in biological data visualization, as well as bioinformatics and biology researchers in state-of-the-art visualization research. The symposium also served as a platform for researchers in biology and bioinformatics to share pressing visualization challenges and potential solutions in their fields, to initiate interdisciplinary collaborations and to provide an outlet and training ground for young and freshly minted visualization researchers with a keen interest in problems of biology.

For the BioVis 2011 Symposium, a new data visualization/analysis contest was inaugurated. The main goals of the contest were: the development of a better-informed visualization community, provided with deeper domain-specific intuition into the actual issues of interest to the biology and bioinformatics communities; a better-tooled biological community, provided with enhanced application software specifically adjusted to meet their analysis needs; and, finally, a mechanism to strongly promote fundable peer collaborations between visualization and bioinformatics/biology researchers. This contest was focused on specific biological problem domains, based on realistic domain data and domain questions. For BioVis 2011, the contest involved the analysis of expression Quantitative Trait Locus (eQTL) data. Judging for the contest entries was conducted by a panel of experts, including members from both the bioinformatics/biology and the visualization communities.

The procedure for selecting the articles for this Supplement was as follows. A first selection of best papers from the twenty paper contributions to BioVis 2011 was made by the paper chairs, based on the reviewer scores and recommendations and the authors were requested to submit an extended version of their paper with at least 30% additional, unpublished, material. In addition, one paper was invited to discuss the BioVis 2011 contest, summarizing the problem and the contest submissions and discussing the lessons learned, for example, what worked, what didn't, and what seemed to be the biggest challenges in coming to a solution. These papers went through a new reviewing round with two independent reviewers. In agreement with the BMC peer review guidelines, the supplement editors did not review or make acceptance decisions about any manuscript to which they had contributed. Following the reviewing process papers were either accepted after taking into account the recommendations by the reviewers or rejected. As a result, seven papers have been accepted for this Supplement.

The articles in this Supplement cover a wide spectrum of challenging problems in biological data visualization and their solutions. *Heinrich et al*. describe the interactive Hierarchical Aggregation Table (iHAT), which facilitates the visualization of multiple sequence alignments, associated metadata, and hierarchical clusterings. *Smith et al*. present RuleBender, a novel visualization system for the integrated visualization, modeling and simulation of rule-based intracellular biochemistry. In the same spirit, *Strobelt et al*. present HiTSEE (High-Throughput Screening Exploration Environment), a visualization tool for the analysis of large chemical screens used to examine biochemical processes. *Paterson et al*. describe VIPER (Visual Pedigree Explorer), a software tool for pedigree visualization that integrates an inheritance-checking algorithm with a novel space-efficient pedigree visualization. *Livengood et al*. present a system that enables interactive comparative visualization and analytics of metabolomics data obtained by two-dimensional gas chromatography-mass spectrometry. *Mayerich et al*. present NetMets, a method for quantifying and visualizing errors in biological network segmentation. Finally, *Bartlett et al*. present and discuss the eQTL biological data visualization challenge conducted as the BioVis 2011 contest. This is a tremendously important biological grand challenge domain with no extant solutions. The authors provide a thought-provoking perspective on the complex relations between the biology, bioinformatics, and visualization domains. They discuss the contest entries submitted to BioVis 2011, present the lessons learned, and conclude with open questions for the future.

We hope that the collection of papers in this Supplement will enlighten the reader about the intense activity in the challenging field of biological data visualization, and will encourage and stimulate the interaction and collaboration between researchers from the biology, bioinformatics, and visualization communities.

## Competing interests

The authors declare that they have no competing interests.

